# Oral Administration of Alkylglycerols Differentially Modulates High-Fat Diet-Induced Obesity and Insulin Resistance in Mice

**DOI:** 10.1155/2013/834027

**Published:** 2013-06-20

**Authors:** Mingshun Zhang, Shuna Sun, Ning Tang, Wei Cai, Linxi Qian

**Affiliations:** ^1^Xinhua Hospital, Shanghai Institute for Pediatric Research, Shanghai Jiao Tong University, School of Medicine, Shanghai 200092, China; ^2^Department of Immunology, Nanjing Medical University, Nanjing 210029, China; ^3^Fudan Children's Hospital, Fudan University, School of Medicine, Shanghai 201102, China; ^4^Shanghai Key Laboratory of Pediatric Gastroenterology and Nutrition, Shanghai 200092, China

## Abstract

Alkylglycerols (AKGs) from shark liver oil (SLO) were demonstrated to have strong potency to stimulate immune response. However, no study has been conducted on the effects of AKGs on diet-induced obesity and metabolic inflammatory disorder. The purpose of the present study was to investigate the effect of two AKGs isoforms on obesity and insulin resistance in mice fed high-fat (HF) diet. Forty-eight C57BL/6 mice were divided into normal, HF, HF + 20 mg/kg selachyl alcohol (SA), HF + 200 mg/kg SA, HF + 20 mg/kg batyl alcohol (BA), and HF + 200 mg/kg BA groups. Body weight, fasting glucose, lipids, insulin and leptin levels, serum IL-1*β*, and TNF-**α** levels were compared among different groups. Our results showed that high-dose SA decreased body weight, serum triglyceride, cholesterol, fasting glucose level, insulin level, and serum leptin level of the HF fed mice, while high-dose BA increased fasting insulin level of the HF fed mice. Pretreatment of primary adipocytes with 10 **μ**M SA or BA differentially modulates LPS-mediated MAPK and NF-**κ**B signaling. Our study demonstrated that oral administration of AKGs has differential effects on HF-induced obesity and metabolic inflammatory disorder in mice.

## 1. Introduction

Obesity has become a common public health issue with a cluster of metabolic abnormalities. The incidence of obesity-related chronic diseases is increasing rapidly worldwide [1]. Evidence has accumulated indicating that obesity is closely associated with a state of systematic, low-grade inflammation characterized by activation of inflammatory signaling pathways and abnormal cytokine production in adipose tissue [2, 3]. The cytokines produced by adipocytes include several inflammatory markers such as interleukin (IL)-6, tumor necrosis factor (TNF)-*α*, and monocyte chemoattractant protein (MCP)-1 [4]. These cytokines are elevated in patients with obesity and insulin resistance and are highly associated with the development of cardiovascular diseases and type 2 diabetes mellitus.

Recently, dietary supplements have been used for prevention of obesity and diabetes mellitus due to their high compliance and low toxicity. Shark liver oil (SLO), a well-known dietary supplement, contains alkylglycerols (AKGs), squalene, and essential fatty acids [5]. It has recently been shown that SLO has various pharmacological benefits such as chemoprotective properties against reactive oxygen species as well as anti-inflammatory, antibacterial, antifungal, and anticancer potency [6]. AKGs, the major component of SLO, are glycerol ether lipids that have structural characteristics of an ether linkage between fatty acid and *α*-position of the glycerol backbone. According to the fatty acid chain length and the number of double bonds, several derivatives of AKGs have been identified. They include such substances as batyl alcohol (BA), chimyl alcohol (CA), and selachyl alcohol (SA) [7]. SA, the predominant component of bioactive AKGs in the SLO (accounting for 59.4%), contains an unsaturated bond in the long hydrocarbon chain (18C:1). CA and BA, which are saturated in their hydrocarbon chains (16C:0 CA, 18C:0 BA), account for a minor proportion of SLO (9.1% CA, 2.8% BA) [6]. AKGs are also found in immune organs such as bone marrow and spleen, indicating their important role in human immune activity [8]. AKGs mainly function by stimulating immune response to enhance the human defense against inflammation [9]. AKGs can also be applied to treat leukemia and solid tumor as well [10]. It was demonstrated that AKGs can inhibit the growth, vascularization, and dissemination of lung carcinoma tumors in mice [11, 12]. 

The antidiabetic effects of various bioactive food components have gained widespread attention. However, it was also demonstrated that some nutrients such as selenium have side-effect on energy metabolism if they are supplemented inappropriately [13]. AKGs have been shown to have capability of activating cytotoxic macrophages leading to an enhanced phagocytosis and elevating Th-1 cytokines such as TNF-*α* which are required for macrophage activation [14]. Adipose tissue macrophages play a key role in obesity-induced inflammation and insulin resistance [15]. However, no study has been conducted on the effects of AKGs on diet-induced obesity and metabolic inflammatory disorder. It is interesting to explore how AKGs affect energy metabolism if consumed daily as a nutrition supplement. Therefore, we examined the effect of AKGs on lipopolysaccharide- (LPS-) mediated insulin resistance and induction of inflammatory genes in high-fat (HF) fed mice. 

## 2. Materials and Methods

### 2.1. Chemicals

SA was purchased from NIKKO Chemicals (Tokyo, Japan). BA was purchased from Bachem (Bubendorf, Switzerland). Escherichia coli LPS 0111:B6 was purchased from Sigma-Aldrich (St. Louis, MO). Glucose, cholesterol, and triglyceride kits were obtained from Kinghawk Pharmaceutical (Beijing, China). Insulin, leptin, IL-1β, and TNF-*α* ELISA kits were purchased from R&D systems (Minneapolis, MN). Antiphospho- (Thr183/Tyr185) and total JNK, antiphospho- (Thr202/Tyr204) and total ERK, and anti-I*κ*B*α* were purchased from Santa Cruz (Santa Cruz, CA). All other chemical reagents used in the present study were of analytical grade. 

### 2.2. Animals and Facilities

The study was approved by the Animal Ethics Committee of Xinhua Hospital. Forty-eight, 4-week old male C57BL/6 mice were purchased from SLAC Laboratories (Shanghai, China). All mice were housed in stainless steel cages with bedding (6 mice/cage). Sufficient bedding was used to keep mice dry and clean. All the mice were exposed to a 12-hour light and dark cycle. Frequent bedding changes and cage cleaning were performed as often as necessary.

### 2.3. Animal Study Design

After arrival, mice were acclimatized for 4 days. After acclimatation, forty-eight mice were randomly divided into six groups of 8 mice each. Both normal chow and high-fat diets were purchased from Shanghai Slac Laboratory Animal Co., Ltd. Normal chow diets contained 20.5% crude protein, 4.62% crude fat, 52.5% nitrogen-free extract, and 4.35% crude fibers (total calories 3.45 Kcal/g, 12% calories in fat). High-fat diets contained 18.8% crude protein, 16.2% crude fat, 45.2% nitrogen-free extract, and 3.98% crude fibers (total calories 3.79 Kcal/g, 38% calories in fat) [16]. For 8 weeks, groups 1 and 2 received the normal diets (ND) and high-fat diets (HF), respectively; groups 3 and 4 were fed the HF supplemented with 20 and 200 mg/kg SA, respectively; groups 5 and 6 were fed the HF supplemented with 20 and 200 mg/kg BA, respectively. Body weight was monitored weekly. At the end of the experiment, blood samples were collected after overnight fasting. Following 4 days recovery, all groups were fasted for 5 hours and then challenged with 100 ng LPS intraperitoneally. After 2 hours, animals were then euthanized and blood samples, liver, and epididymal fat were collected. Liver tissues and visceral adipose were immediately weighted after removal [17]. Serum was isolated by centrifugation at 1500 g at 4°C for 10 min and stored at −80°C until it was used for blood biochemical assays. 

### 2.4. Culturing of Primary Adipocytes

Abdominal white adipose tissue was obtained from 4- to 5-week-old, wild-type mice. After blood washing, the adipose tissues were minced and digested with 1 mg/mL collagenase type I (Sigma-Aldrich, St. Louis, MO) for 30 min at 37°C. Cells were filtered through 200-*μ*m pore size nylon meshes. The stromal vascular cells (SVCs) were separated from adipocytes by centrifugation and washed with DMEM (Invitrogen, Carlsbad, CA) supplemented with 10% fetal bovine serum (FBS). SVCs were plated and propagated to confluence in DMEM supplemented with 10% FBS, 50 *μ*g/mL streptomycin, and 50 U/mL penicillin [18]. After attachment, the medium was replaced by induction medium containing 10 *μ*g/mL insulin (INS), 1 *μ*m dexamethasone (DEX), and 0.5 mm 3-isobutyl-1-methylxanthine (MIX) with 10% FBS and continued differentiation for 12 days. On day 12, cultures were pretreated with DMSO vehicle, or different concentrations of SA or BA for 24 hrs, and then treated with 10 *μ*g/L LPS for 6 hrs. 

### 2.5. Immunoblotting Analysis

Following treatment, cultures were harvested and protein was extracted with RIPA buffer. Immunoblotting analysis was performed as described previously [19]. 

### 2.6. Biochemical Analysis

Concentrations of insulin, leptin, IL-1β, and TNF-*α* were measured using ELISA kit. Glucose, total cholesterol, and triglyceride were tested using enzymatic methods. Homeostatic Model Assessment-Insulin Resistance (HOMA-IR) was calculated from glucose and insulin concentrations (fasting glucose (mmol/L) × fasting insulin (*μ*U/mL)/22.5) [20]. 

### 2.7. Statistical Analysis

Data are shown as means with their standard errors. Statistical significance was evaluated using one-way ANOVA followed by Duncan's multiple range test. *P* value < 0.05 was considered statistically significant.

## 3. Results

### 3.1. Effects of AKGs Diets on Body and Organ Weights

Daily food intake during the experimental period was not significantly different among groups. No changes of end-point body weight and net weight gain were observed between HF diet group and low-dose (20 mg/kg) AKGs (SA or BA) supplemented groups during the 60-day period. High-dose SA (200 mg/kg) supplementation significantly decreased the end-point body weight and net weight gain of the HF fed mice during the 60-day dietary intervention. Weights of epididymal white adipose tissue and liver were compared among the different dietary treatments. Epididymal fat was significantly decreased as percent body weight in mice that received 200 mg/kg SA supplementation (Table 1). 

### 3.2. Effects of AKGs Diets on Serum Triglyceride and Cholesterol

**Figure 1:**
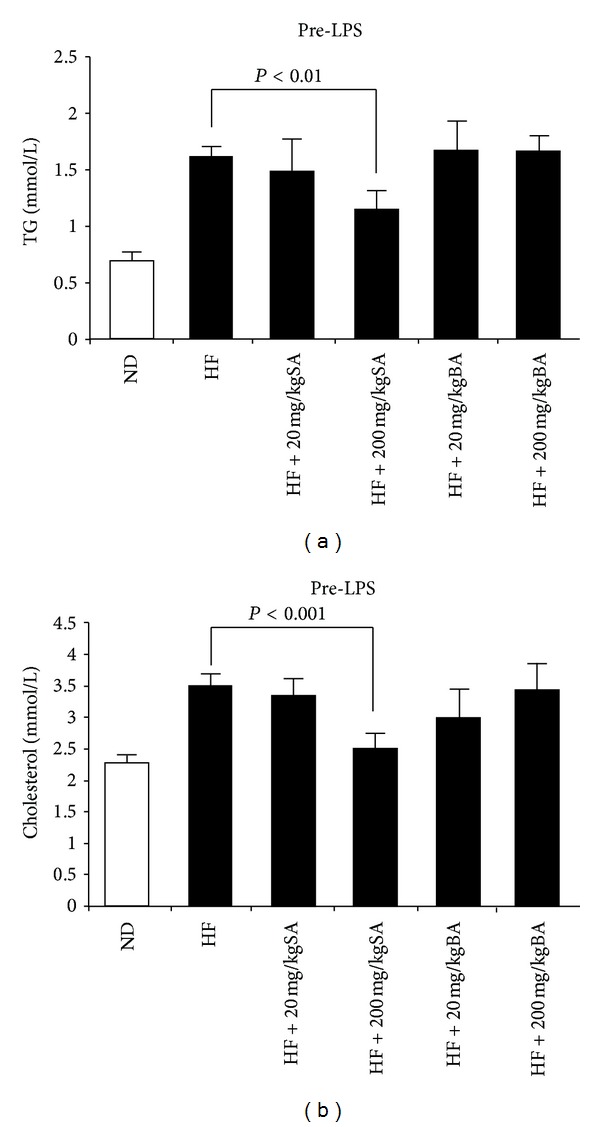
The effect of selachyl alcohol (SA) or batyl alcohol (BA) supplementation on serum triglycerides (a) and cholesterol (b). Data were presented as mean ± SE. Significant differences were tested with two-way ANOVA. Only significant comparisons are presented.

There was a significant (110%) increase in the serum triglyceride level of the HF group compared with the ND group, whereas a 25% decrease of serum triglyceride was observed in the 200 mg/kg SA group relative to the HF group (*P* < 0.01) (Figure 1(a)). HF feeding caused a significant (50%) increase in the total cholesterol level. 200 mg/kg SA treatment; however, had reduced the serum cholesterol level by 30% as compared to the HF group (*P* < 0.001) (Figure 1(b)). There was no significant difference in triglyceride or cholesterol level between the HF group and the HF plus low-dose SA group. No significant change in triglyceride or cholesterol level was observed in HF plus BA diet group as compared with HF group.

### 3.3. Effects of AKGs Diets on Glucose, Insulin, and HOMA-IR

To investigate the impact of different AKGs supplemented HF diets in comparison with HF diet on glucose metabolism, we examined blood glucose concentrations before and after LPS challenge in different dietary groups. HF diet group had significantly higher fasting blood glucose concentration as compared to ND diet group (9.84 mmol/L versus 7.62 mmol/L; *P* < 0.001) (Table 2). Fasting blood glucose was significantly lower in mice fed HF diet plus 20 or 200 mg/kg SA than that in mice fed HF diet (*P* < 0.001). After intraperitoneal injection of LPS, a drop of blood glucose concentration was observed in all groups. HF diet plus 200 mg/kg SA caused lower blood glucose concentration after LPS challenge as compared to HF diet group (5.05 mmol/L versus 5.71 mmol/L; *P* < 0.05) (Table 2). 

The impact of different forms of AKGs on insulin resistance induced by HF diet was also assessed. The plasma insulin concentrations were examined before and after LPS challenge in different dietary groups. As expected, HF diet group had significantly higher fasting insulin concentrations as compared to ND diet group (27.81 *μ*IU/mL versus 13.49 *μ*IU/mL; *P* < 0.001) (Table 2). It was noted that fasting insulin concentration was decreased in mice fed HF diet plus 200 mg/kg SA relative to mice fed HF diet (20.92 *μ*IU/mL versus 27.81 *μ*IU/mL; *P* < 0.05), whereas insulin concentration was increased in mice fed HF diet plus 200 mg/kg BA (*P* < 0.05). After intraperitoneal injection of LPS, mice fed HF plus 200 mg/kg SA showed lower insulin increase compared to mice fed HF diet (22.92 *μ*IU/mL versus 37.13 *μ*IU/mL; *P* < 0.05). No effects of low-dose AKGs (SA or BA) supplementation were observed on fasting insulin concentrations at pre- and post-LPS challenge (Table 2). The HOMA-IR score was calculated from fasting blood glucose and insulin concentration to assess whether AKGs diet protected mice from insulin resistance. In fact, mice that received 20 or 200 mg/kg SA supplementation had significantly lower HOMA-IR scores (*P* < 0.001), however, mice received 200 mg/kg BA supplementation had significantly higher HOMA-IR scores as compared to mice that received HF diet at pre-LPS challenge (*P* < 0.05). After intraperitoneal injection of LPS, only 200 mg/kg SA supplementation showed significant protective effect on insulin resistance which was indicated by HOMA-IR scores (*P* < 0.005) (Table 2).

### 3.4. Effect of AKGs on Serum Cytokines and Leptin

The proinflammatory cytokines such as IL-1β and TNF-*α* were elevated after LPS challenge. Interestingly, high-dose SA or BA showed differential effects on serum IL-1β and TNF-*α* production after 100 ng LPS challenge (Table 3). 200 mg/kg SA supplementation suppressed serum IL-1*β* and TNF-**α** level induced by LPS challenge (*P* < 0.05), whereas 200 mg/kg BA supplementation significantly enhanced serum IL-1β and TNF-**α** level induced by LPS challenge (*P* < 0.05). However, there was no significant effect on IL-1*β* and TNF-**α** response to LPS challenge by low-dose AKGs (SA or BA) supplementation. 

As for serum leptin, mice that received HF diet had significantly higher serum leptin concentration compared to those received ND diet (16.55 ng/mL versus 7.26 ng/mL; *P* < 0.05). We found that serum leptin concentration was significantly decreased in mice that received 200 mg/kg SA supplementation as compared to mice that received HF diet at pre-LPS challenge (13.57 ng/mL versus 16.55 ng/mL; *P* < 0.05) (Table 3).

### 3.5. Effect of AKGs on LPS-Mediated MAPK and NF-*κ*B Activation

**Figure 2:**
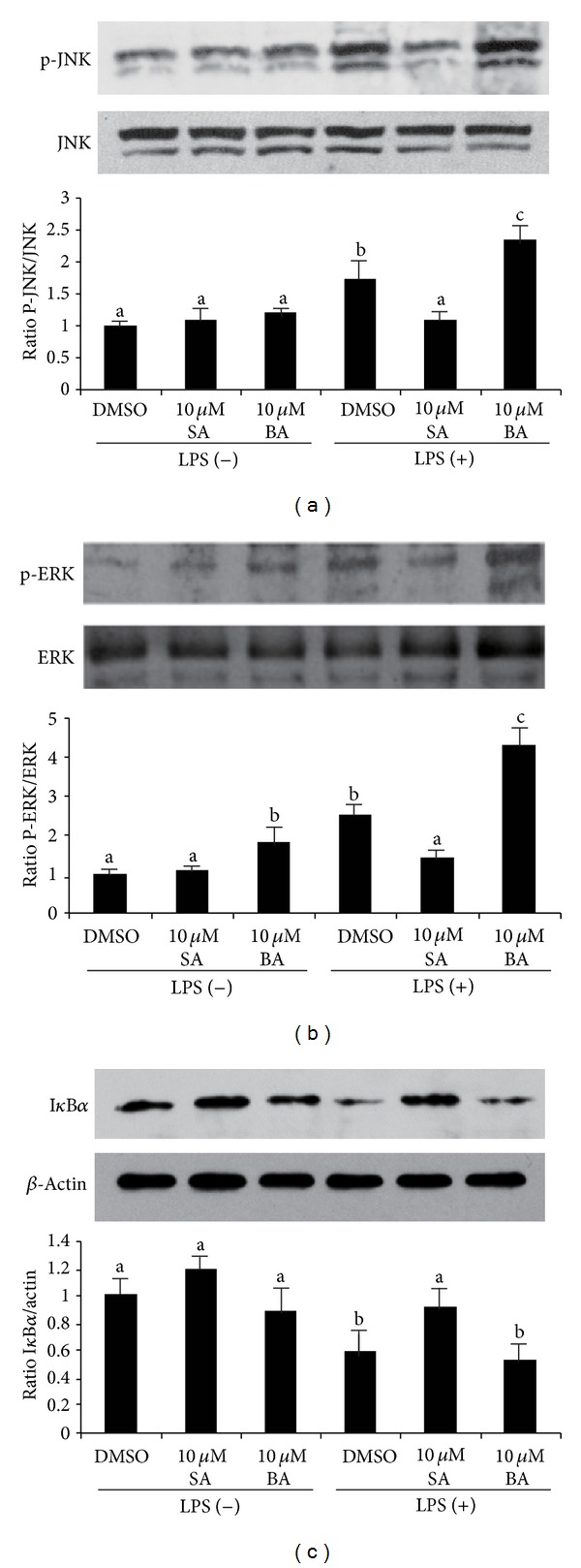
The effect of selachyl alcohol (SA) or batyl alcohol (BA) on TLR-4 signaling activated by LPS in mice primary adipocytes. Newly differentiated cells were pretreated with DMSO vehicle (−) or 10 *μ*M SA or 10 *μ*M BA for 24 h and then treated with 10 *μ*g/L LPS for 6 h. (a) Phosphorylation of JNK (P-JNK-to-JNK ratio). (b) Phosphorylation of ERK (P-ERK-to-ERK ratio). (c) Expression of I*κ*B*α* (I*κ*B*α*-to-actin ratio). Levels of protein were measured by western blotting. Data are a mean of ± SE and corrected for loading. Significant differences between each group are indicated by different letters.

Given the role of MAPK pathway in inducing inflammatory gene expression via Toll-like-receptor- (TLR-) 4 activation, we examined the effects of AKGs on MAPK phosphorylation. Pretreatment of mice adipocyte cultures with 10 *μ*M SA modestly decreased LPS-mediated phosphorylation of JNK and ERK (Figure 2). However, pretreatment of mice adipocyte cultures with 10 *μ*M BA increased LPS-mediated phosphorylation of JNK and ERK. In the absence of LPS, AKGs treatment did not enhance the MAPK activation. Because the activation of NF-*κ*B also plays a crucial role in the transcriptional activation of inflammation-responsive genes, the effects of AKGs on NF-*κ*B activation were examined by detecting I*κ*B*α* degradation via immunoblot. We found that the pretreatment with SA attenuated I*κ*B*α* degradation by LPS (Figure 2). However, BA did not present effect on LPS-induced NF-*κ*B activation in our experiments.

## 4. Discussion

SLO has been widely used in the past years in the Scandinavian medicine because of its properties as immunity boosters and a remedy against radiation therapy and cancer [21]. AKGs are the major components in SLO which could stimulate immunity both *in vitro* and *in vivo* [22, 23]. Daily consumption of AKGs-rich SLO showed benefits to the immune system. Despite widespread intake of AKGs, safety studies on AKGs extract were poor. The acute and repeated (28 days) oral toxicity has been evaluated for oral AKGs administration in rats at doses of 200 and 1000 times the maximum recommended dose in humans [24]. In that study, AKGs administration showed no adverse effects on mortality at either acute or subchronic dose. However, the correlation between long-term AKGs supplement and HF-induced obesity has not been shown. In previous studies, AKGs were demonstrated to have potency to activate cytotoxic macrophages and increase humoral immune response [22]. AKGs could also stimulate the IL-12 and IFN-gamma production and elicit Th1 response [25, 26]. As we know, adipose infiltrated macrophage and secreted cytokines play important roles in obesity and insulin resistance [27–29]. Thus, the potency of AKGs to stimulate immunity spurred our interest to examine the AKGs effect on HF-induced obesity and insulin resistance.

Studies have shown that HF diets induce adipose tissue inflammation and stimulate TLR-4 expression [30]. TLR-4, a subclass of the TLR family, plays a critical role in activating innate immune and inflammation response in mammals by recognizing bacterial LPS [31, 32]. Activation of TLR-4 in adipocytes leads to the activation of MAPK and NF-*κ*B signaling pathways, and induction of many inflammatory cytokines [33, 34]. These cytokines are involved in inducing glucose intolerance, insulin resistance, and infiltration of macrophages into adipose tissue. Recent study showed that TLR-4 responds to nonbacterial ligands such as fatty acids [35–37]. It was demonstrated that saturated fatty acids such as lauric acid and palmitic acid are able to induce cyclooxygenase-2 (COX-2) expression; however, unsaturated fatty acids such as docosahexaenoic acid (DHA) and eicosapentaenoic acid (EPA) are able to inhibit saturated fatty acid-induced COX-2 expression [38]. In present study, we demonstrated that AKGs with saturated chain increased LPS-mediated activation of the MAPK signaling, which could cause the expression of inflammatory genes and insulin resistance in adipocytes. AKGs with unsaturated chain decreased LPS-mediated activation of the MAPK and NF-*κ*B signaling, thus ameliorated insulin resistance. However, both AKGs with saturated and unsaturated chain did not activate MAPK or NF-*κ*B signaling in the absence of LPS. Accordingly, it was indicated that both AKGs with saturated chain and unsaturated chain modulated TLR-4 signaling and insulin response in the hyperinflammatory environment such as high-fat diet feeding or LPS treatment but did not show direct effect in the hypoinflammatory environment. This result was also noticed by Ocaña et al., who showed that BA activated the expression of IL-1β and IL-6 genes only in TNF-*α*-induced adipocytes but not in the nonstimulated cells [39]. 

Several studies have demonstrated that different forms of AKGs have differential biological effects. The unsaturated AKGs with 16 or 18 carbon alkyl chains showed strong anti-tumor and antimetastasis activities in mice model [40]. By contrast, the saturated AKGs with 16 or 18 carbon alkyl chains showed less antitumor effect or even tumor-promoting activity. Previous studies also demonstrated that proinflammatory responses were enhanced by most saturated fatty acids but reduced by most unsaturated fatty acids. In metabolic experiments, rats fed saturated fatty acids had higher triacylglycerol, cholesterol, and low-density lipoprotein cholesterol, whereas rats fed unsaturated fatty acids had lower triacylglycerol, cholesterol, and low-density lipoprotein cholesterol as compared with control rats [41, 42]. Moreover, the increased ratio of P/S (polyunsaturated/saturated) fatty acids was beneficial in depleting white adipose tissue accumulation and improved the metabolic status in diet-induced obese hamster [43]. The controlled clinical trials have also indicated that replacing saturated fat with unsaturated fat was more effective in lowering risk of metabolic disorder than simply reducing total fat consumption [44, 45]. Our studies showed that AKGs had the similar effects on metabolic and inflammatory status as free fatty acids in spite of their ether bond with glycerol. As we know, SLO and human breast milk are both good sources of AKGs, with unsaturated forms as the predominant components. In clinical studies, SLO showed beneficial effect on lipid metabolism in patients with hypertension [46]. Breast milk has also been demonstrated to be protective against the development of childhood overweight and obesity [47]. Thus, we postulated that the predominant unsaturated AKGs in SLO and breast milk would neutralize the adverse effects of saturated AKGs on metabolism and play a prominent role of preventing metabolic syndrome. 

Increasing studies have indicated that dietary supplementation of oils extracted from marine species can alter the metabolic and immunologic status which is highly associated with the incidence of obesity and type 2 diabetes. Different marine oils have distinct lipid profiles, which result in potential differences in their effects on inflammation and metabolism. Fish oil, the typical marine oil product, was demonstrated to have protective effect on LPS-induced inflammation and insulin resistance [17]. It was also shown that dietary fish oil supplementation could reduce body weight gain in HF-induced obese mice [48]. The anti-inflammation and antiobesity effects of fish oil were attributed to its richness of (*n* − 3) PUFA [49]. SLO, which was also rich of (*n* − 3) PUFA, was known to contain high proportion of squalene and AKGs. Both squalene and AKGs have potent immunological activity, thus eliciting people's interest of investigating their impact on metabolic balance. Squalene has already been demonstrated to be able to elevate the body weight and serum cholesterol in high-fat fed hamsters due to its involvement in hepatic cholesterol metabolism [50]. However, little is known about the association between AKGs intake and metabolic alteration. Our results showed that AKGs differentially modulated HF-induced obesity and insulin resistance in mice, which gave us indication that more attention should be paid to the impact of these immune-stimulating chemicals on metabolism. 

Actually, it has already been noticed that oral SLO intake has adverse effect on liver function if supplemented at high dosage [51]. Therefore, it is important to use AKGs in animal experiment at the dosage comparable to that of human supplementation for nutritional purpose. The Biomare Immuno which is manufactured by Hankintatukku (Finland) contains 60 mg AKGs per capsule. On the label, it recommends six capsules daily, which equals to 7.2 mg AKGs/kg^weight^ (60 mg × 6/50 kg^weight^) provided that a male adult weighs 50 kg on average. In the present study, AKGs at 20 mg/kg^diet^ and 200 mg/kg^diet^ dietary supplementation corresponded to 4 mg/kg^weight^ (20 mg/kg^diet^ × 0.005 kg^diet^/0.025 kg^weight^) and 40 mg/kg^weight^ (200 mg/kg^diet^×0.005 kg^diet^/0.025 kg^weight^), respectively, assuming that a male adult mouse weighs 25 g and expends 5 g diet on average. Thus, the amount of 20 mg/kg^diet^ AKGs dietary supplement was comparable to that commonly consumed by a SLO-supplement consumer. The results obtained from rodents showed that the 20 mg/kg^diet^ dietary AKGs supplementation did not have significant effect on body weight but only showed minor effect on glucose metabolism. Nevertheless, 200 mg/kg^diet^ dietary AKGs supplementation showed obvious effects not only on body weight but also on lipid, glucose metabolism, and pro-inflammatory cytokine production. Although these results were not directly applicable to humans, they provided some implications for individuals who routinely consume AKGs supplement or AKGs-rich SLO. Taken together, our results provided novel insights into differential effects of saturated- and unsaturated-chain AKGs on adipose tissue inflammation, which suggested that routine consumption of SLO consisting of different isoforms of AKGs is safe at normal dosage and shows balanced effect on obesity and insulin resistance.

## 5. Conclusion

Collectively, these data demonstrate that the unsaturated AKGs (SA) have potency to decrease the HF-induced obesity at high dosage and ameliorate insulin resistance at normal or high dosage. The saturated AKGs (BA) can increase insulin resistance at high dosage. Our data also suggest that AKGs show differential effects on LPS-induced inflammation in adipocytes. The effect of SLO rich for AKGs on adipose metabolism and insulin response should be evaluated in the future, to warrant supplementing SLO products at a safe dosage and prevent its potential hazardous effects on metabolism. 

## Figures and Tables

**Table 1:** The effect of selachyl alcohol (SA) or batyl alcohol (BA) supplementation on body weights, tissue weights, and food consumption.

		Treatment^1^							
							*P* value^6^	*P* value	*P* value
Item	ND	HF	HF + 20 mg/kg SA	HF + 200 mg/kg SA	HF + 20 mg/kg BA	HF + 200 mg/kg BA	(HF)	(SA)	(BA)
Initial body wt^2^ (g)	14.05 ± 0.18^5^	14.27 ± 0.21	14.38 ± 0.23	14.36 ± 0.27	14.22 ± 0.29	14.33 ± 0.23	0.299	0. 943	0.950
End-point body wt^3^ (g)	25.92 ± 0.56	35.21 ± 0.57	36.48 ± 0.59	32.80 ± 0.54*	36.35 ± 0.44	36.17 ± 0.39	<0.001	<0.001	0.087
Net weight gain^4^ (g)	11.87 ± 0.63	20.93 ± 0.51	22.10 ± 0.67	18.43 ± 0.57*	22.12 ± 0.55	21.83 ± 0.54	<0.001	<0.001	0.062
Epididymal fat^ 3^(%)	1.76 ± 0.29	2.39 ± 0.09	2.20 ± 0.05	2.02 ± 0.10*	2.48 ± 0.08	2.64 ± 0.07	<0.001	0.019	0.130
Liver weight^3^ (%)	3.86 ± 0.04	3.29 ± 0.05	3.42 ± 0.07	3.49 ± 0.08	3.37 ± 0.02	3.18 ± 0.03^†^	<0.001	0.150	0.011
Food intake (g/mouse/day)	2.80 ± 0.05	2.64 ± 0.04	2.57 ± 0.04	2.58 ± 0.05	2.64 ± 0.06	2.69 ± 0.05	0.005	0.563	0.729

^1^ND: normal diet; HF: high-fat diet; SA: selachyl alcohol; BA: batyl alcohol.^2^Measured before dietary intervention. ^3^Measured after 8-week feeding period. ^4^Difference between end-point body weight and initial body weight. ^5^Values are mean ± SEM. Means with a mark (*) in HF + 20 mg/kg  SA or HF + 200 mg/kg SA group differ significantly from the HF diet group. Means with a mark (^†^) in HF + 20 mg/kg BA or HF + 200 mg/kg BA group differ significantly from the HF diet group. ^6^ *P* value (HF) indicates the effect of HF treatment. *P* value (SA) indicates the effect of different doses of SA supplements on HF fed mice. *P* value (BA) indicates the effect of different doses of BA supplements on HF fed mice.

**Table 2:** The effect of selachyl alcohol (SA) or batyl alcohol (BA) supplementation on serum glucose, insulin, and Homeostatic Model Assessment-Insulin Resistance (HOMA-IR) before and after 100 ng lipopolysaccharide (LPS) challenge.

		Pre-LPS^1^							
							*P* value^4^	*P* value	*P* value
Item	ND^2^	HF	HF + 20 mg/kg SA	HF + 200 mg/kg SA	HF + 20 mg/kg BA	HF + 200 mg/kg BA	(HF)	(SA)	(BA)
Glucose (mmol/L)	7.62 ± 0.30^3^	9.84 ± 0.34	8.69 ± 0.20*	7.47 ± 0.29*	10.03 ± 0.37	10.06 ± 0.54	<0.001	<0.001	0.761
Insulin (*μ*IU/mL)	13.49 ± 1.14	27.81 ± 1.85	25.05 ± 2.03	20.92 ± 1.5*	25.33 ± 2.73	34.41 ± 2.03^†^	<0.001	0.043	0.025
HOMA-IR	4.63 ± 0.54	12.11 ± 0.78	9.71 ± 0.86*	7.00 ± 0.69*	11.12 ± 1.02	15.39 ± 1.17^†^	<0.001	<0.001	0.017

		Post-LPS							
Glucose (mmol/L)	6.19 ± 0.30	5.71 ± 0.16	5.73 ± 0.21	5.05 ± 0.22*	5.16 ± 0.15	5.88 ± 0.30	0.182	0.038	0.062
Insulin (*μ*IU/mL)	21.20 ± 3.35	37.13 ± 2.86	33.03 ± 3.65	22.99 ± 3.08*	33.86 ± 2.42	39.92 ± 2.33	<0.005	0.018	0.246
HOMA-IR	5.71 ± 0.79	9.48 ± 0.91	8.35 ± 0.91	5.11 ± 0.70*	7.80 ± 0.62	10.62 ± 1.08	0.008	0.004	0.096

^1^Serum glucose and insulin were measured before LPS challenge (Pre-LPS) and 2 hours following 100 ng LPS challenge (Post-LPS). ^2^ND: normal diet; HF: high-fat diet; SA: selachyl alcohol; BA: batyl alcohol. ^3^Values are mean ± SEM. Means with a mark (*) in HF + 20 mg/kg SA or HF + 200 mg/kg SA group differ significantly from the HF diet group. Means with a mark (^†^) in HF + 20 mg/kg BA or HF + 200 mg/kg BA group differ significantly from the HF diet group. ^4^ *P* value (HF) indicates the effect of HF treatment. *P* value (SA) indicates the effect of different doses of SA supplements on HF fed mice. *P* value (BA) indicates the effect of different doses of BA supplements on HF fed mice.

**Table 3:** The effect of selachyl alcohol (SA) or batyl alcohol (BA) supplementation on serum cytokines and leptin before and after 100 ng lipopolysaccharide (LPS) challenge.

		Pre-LPS^1^							
							*P* value^4^	*P* value	*P* value
Item	ND^2^	HF	HF + 20 mg/kg SA	HF + 200 mg/kg SA	HF + 20 mg/kg BA	HF + 200 mg/kg BA	(HF)	(SA)	(BA)
IL-1β (pg/mL)	2.3 ± 0.19^3^	8.8 ± 0.70	8.25 ± 1.09	7.1 ± 0.82	8.18 ± 0.59	10.47 ± 1.37	<0.001	0.404	0.074
TNF-*α* (pg/mL)	4.83 ± 0.56	5.83 ± 0.29	5.5 ± 0.44	5.95 ± 0.25	5.07 ± 0.39	5.68 ± 0.36	0.138	0.625	0.289
Leptin (ng/mL)	7.26 ± 0.50	16.55 ± 0.60	16.18 ± 0.59	13.57 ± 0.49*	16.52 ± 0.42	16.13 ± 0.55	<0.001	0.005	0.837

		Post-LPS							
IL-1β (pg/mL)	100.85 ± 9.2	196.2 ± 15.4	187.1 ± 10.1	149.6 ± 6.7*	204.5 ± 10.8	253.1 ± 8.7^†^	<0.001	0.016	0.005
TNF-*α* (pg/mL)	231 ± 8.78	347 ± 10.23	320.75 ± 13.06	288.87 ± 9.49*	370.25 ± 14.44	476.79 ± 19.28^†^	<0.001	0.004	<0.001
Leptin (ng/mL)	9.18 ± 0.32	18.16 ± 0.83	16.74 ± 0.76	16.27 ± 0.78	16.37 ± 0.8	16.81 ± 0.68	<0.001	0.063	0.238

^1^Serum cytokines and leptin were measured before LPS challenge (Pre-LPS) and 2 hours following 100 ng LPS challenge (Post-LPS). ^2^ND: normal diet; HF: high-fat diet; SA: selachyl alcohol; BA: batyl alcohol. ^3^Values are mean ± SEM. Means with a mark (*) in HF + 20 mg/kg SA or HF + 200 mg/kg SA group differ significantly from the HF diet group. Means with a mark (^†^) in HF + 20 mg/kg BA or HF + 200 mg/kg BA group differ significantly from the HF diet group. ^4^ *P* value (HF) indicates the effect of HF treatment. *P* value (SA) indicates the effect of different doses of SA supplements on HF fed mice. *P* value (BA) indicates the effect of different doses of BA supplements on HF fed mice.

## Abbreviations

AKGs: alkylglycerols
SA: Selachyl alcohol
BA: Batyl alcohol
HF: High fat
ND: Normal diets
LPS: Lipopolysaccharides
IL: Interleukin
TNF: Tumor necrosis factor
SLO: Shark liver oil
SVCs: Stromal vascular cells
HOMA-IR: Model assessment-insulin resistance
TLR: Toll-like-receptor
PUFAs: Polyunsaturated fatty acids.
